# Disability risk prediction model based on machine learning among Chinese healthy older adults: results from the China Health and Retirement Longitudinal Study

**DOI:** 10.3389/fpubh.2023.1271595

**Published:** 2023-11-09

**Authors:** Yuchen Han, Shaobing Wang

**Affiliations:** School of Public Health, Hubei University of Medicine, Shiyan, Hubei, China

**Keywords:** prediction model, disability, machine learning, older adults, China Longitudinal Study

## Abstract

**Background:**

Predicting disability risk in healthy older adults in China is essential for timely preventive interventions, improving their quality of life, and providing scientific evidence for disability prevention. Therefore, developing a machine learning model capable of evaluating disability risk based on longitudinal research data is crucial.

**Methods:**

We conducted a prospective cohort study of 2,175 older adults enrolled in the China Health and Retirement Longitudinal Study (CHARLS) between 2015 and 2018 to develop and validate this prediction model. Several machine learning algorithms (logistic regression, k-nearest neighbors, naive Bayes, multilayer perceptron, random forest, and XGBoost) were used to assess the 3-year risk of developing disability. The optimal cutoff points and adjustment parameters are explored in the training set, the prediction accuracy of the models is compared in the testing set, and the best-performing models are further interpreted.

**Results:**

During a 3-year follow-up period, a total of 505 (23.22%) healthy older adult individuals developed disabilities. Among the 43 features examined, the LASSO regression identified 11 features as significant for model establishment. When comparing six different machine learning models on the testing set, the XGBoost model demonstrated the best performance across various evaluation metrics, including the highest area under the ROC curve (0.803), accuracy (0.757), sensitivity (0.790), and F1 score (0.789), while its specificity was 0.712. The decision curve analysis (DCA) indicated showed that XGBoost had the highest net benefit in most of the threshold ranges. Based on the importance of features determined by SHAP (model interpretation method), the top five important features were identified as right-hand grip strength, depressive symptoms, marital status, respiratory function, and age. Moreover, the SHAP summary plot was used to illustrate the positive or negative effects attributed to the features influenced by XGBoost. The SHAP dependence plot explained how individual features affected the output of the predictive model.

**Conclusion:**

Machine learning-based prediction models can accurately evaluate the likelihood of disability in healthy older adults over a period of 3 years. A combination of XGBoost and SHAP can provide clear explanations for personalized risk prediction and offer a more intuitive understanding of the effect of key features in the model.

## 1. Introduction

Currently, there are approximately 900 million people aged 60 years or above worldwide, and this number is expected to double by 2050 (1). As people age, most individuals commonly experience a decline in their health or a gradual loss of the functional ability to perform basic yet valuable daily activities, such as bathing, doing laundry, or eating (2). Functional disability is closely correlated with adverse outcomes, such as a decrease in the quality of life, an increase in hospitalization rates, and mortality risks (3–5). The incidence of disability among older adults has become an increasingly serious public health issue. Extending their healthy lifespan and the ability to live independently without relying on others has become a fundamental social goal (6, 7). Meanwhile, functional decline during aging is dynamic and reversible. In a meta-analysis, 13.7% of older adults showed an improvement in their frailty over an average follow-up period of 3.9 years (8). Therefore, accurately predicting disability risk and developing targeted interventions are crucial for reducing the burden of disability among older adults.

In order to accurately predict the 3-year disability incidence rate in healthy older adults, it is essential to first analyze the factors that are most closely related to disability in older adults. Previous research has identified a series of risk factors for disability in older adults, including advanced age (9), the lack of social support (10), abnormal blood lipids (11), smoking (12), abnormal body mass index (13), slow gait speed (14), depression symptoms (15), and poor memory (16). However, these studies have typically been limited to evaluating the relationship between specific predictive factors and disability rather than conducting a comprehensive analysis of multiple factors. While there have been a few studies that have used machine learning to predict disability risk in older adults (17–19), these studies have been primarily conducted on populations in other countries, and there has been limited investigation of disability risk in older adults in China. However, there are still two main limitations to these studies. First, very few research studies have used machine learning methods to achieve objective variable pre-selection, which may lead to issues such as multicollinearity or overfitting due to the inclusion of too many variables. Second, disability is the result of long-term accumulation. To our knowledge, previous studies have not assessed functional disability status in older adults at baseline, which creates a strong correlation between activity levels at that time and outcomes. To address these limitations, this study used machine learning feature selection methods and selected healthy older adults at baseline in order to construct a disability prediction model for this population in China.

Machine learning methods can be used to extract non-linear and seemingly irrelevant factors that are difficult to find with traditional methods, thereby allowing for more accurate feature selection (20, 21). Machine learning algorithms, including logistic regression, KNN, NB, MLP, random forest, and XGBoost, will be used to develop and evaluate prediction models. However, previous machine learning modeling studies have encountered various issues (22, 23). To establish a more precise and generalizable model, this study employs LASSO regression for feature selection, resampling techniques to address class imbalance, normalization on both training and testing data to avoid data leakage, grid search for hyperparameter tuning to improve model performance, and DeLong test to ensure no overfitting occurs.

This study compares various machine learning algorithms by examining performance metrics, including receiver operating characteristic (ROC) curve area under the curve (AUC), accuracy, sensitivity, specificity, and F1 score, to evaluate the performance of the model. Due to the complex non-linear relationships of some ML algorithms, the model results may be difficult to interpret, leading to the “black box” problem, which limits the practical application of prediction models (24). This study will employ SHapley Additive exPlanations (SHAP) on the best-performing machine learning algorithm models to explain individual predictions for both kernel-based approaches and tree-based models. Compared with other interpretation methods found in prior literature, SHAP has distinct advantages in visualizing complex ML prediction models (25). These benefits make it possible to solve the “black box” problem of complex ML models; however, this advanced model interpretation method has not yet been used to predict the risk of disability in healthy older adults in China.

Therefore, the purpose of this study was to develop and validate a predictive model for the 3-year incidence of disability in a Chinese population of healthy older adults, using six machine learning algorithms, while overcoming the limitations of previous research. In addition, the SHAP method will be used to select the best-performing model for further disability risk prediction and interpretability. The study's findings will contribute to timely and targeted intervention measures, promote healthy aging, reduce health disparities, and guide future research in this field.

## 2. Materials and methods

### 2.1. Data and participants

The data used in this study are from the China Health and Retirement Longitudinal Survey (CHARLS)(26). CHARLS is a longitudinal survey that aims to be a representative of the residents in China aged 45 and older, with no upper age limit. To ensure the adoption of best practices and international comparability or results, CHARLS is harmonized with leading international research studies in the Health and Retirement Study (HRS) model. A stratified (by per capita GDP of urban districts and rural counties) multi-stage (county/district-village/community household) PPS random sampling strategy was adopted. The national baseline survey was conducted in 2011–12, with wave 2 in 2013, wave 3 in 2015, and wave 4 in 2018. In order to ensure sample representativeness, the CHARLS baseline survey covered 150 countries/districts and 450 villages/urban communities across the country, involving 17,708 individuals in 10,257 households, reflecting the mid-aged and older Chinese population collectively.

We included 18,085 participants from the 2015–2018 study wave, 2,175 of whom were eligible for model development and internal validation. None of these participants had a disability in the 2015 survey. The inclusion criteria include the following: (1) participants aged 60 years or older as the United Nations defines this age group as older adults (27); (2) participants with complete responses to ADL and IADL in the 2015–2018 survey, which is an indicator of disability; and (3) participants who did not report any ADL or IADL injuries in the 2015 survey, and the absence of disability at baseline was the baseline condition for the study. The World Health Organization defines health as “the complete state of physical, mental, and social wellbeing” (28). Therefore, the exclusion criteria include the following: (1) participants who reported disability issues, including physical disability, brain injury/intellectual disability, blindness or partial blindness, deafness or partial deafness, and muteness or severe stuttering; (2) participants diagnosed with serious illnesses such as cancer and dementia; and (3) participants diagnosed with emotional, nervous, or psychiatric problems by a doctor.

### 2.2. Research variable

#### 2.2.1. Outcome variable

Disability was assessed by activities of daily living (ADL) and instrumental activities of daily living (IADL) (29, 30). The ADL measured the respondent's ability to perform daily tasks, including dressing, bathing, eating, getting out of bed, using the toilet, and controlling urination and defecation, while the IADL determined the ability to perform instrumental activities, such as doing chores, preparing hot meals, shopping, managing money, making phone calls, and taking medications. The participants' answers were categorized into four responses: (1) No, I do not have any difficulty; (2) I have difficulty but still can do it; (3) Yes, I have difficulty and need help; and (4) I cannot do it. Each ADL/IADL item received scores of 0 if they had no problem performing the activity and 1 if they experienced any difficulty or could not complete the task. We calculated the total score by summing the six items. We classified the ADL/IADL into two categories: (1) no disability (ADL/IADL score = 0) and (2) disability(ADL/IADL score ≥ 1) (31).

#### 2.2.2. Predictive variables

We conducted a preliminary assessment of predictive factors related to disability based on their clinical significance and scientific knowledge and established predictive factors in previous research (32, 33). We selected 43 factors that may be related to the population, including demographic characteristics (age, gender, marital status, and education level); lifestyle and health behaviors (smoking, drinking, sleep duration, naps, and social involvement); laboratory results (white blood cells, hemoglobin, hematocrit, mean corpuscular volume, platelets, triglycerides, creatinine, blood urea nitrogen, high-density lipoprotein cholesterol, low-density lipoprotein cholesterol, total cholesterol, glucose, uric acid, cystatin C, C-reactive protein, and glycated hemoglobin); physical examination results (systolic blood pressure, diastolic blood pressure, pulse, respiratory function, left-hand grip strength, right-hand grip strength, standing balance, walking speed, body mass index, upper arm length, knee length, and waist circumference); satisfaction (health, marriage, children, and life); and memory and depression symptoms measured by the 10-item version of the Center for Epidemiologic Studies Depression Scale (CES-D).

#### 2.2.3. Data collection

The participants' demographic features, lifestyle and health behaviors, and satisfaction were collected by trained staff using questionnaire interviews. The Chinese version of CES-D 10 from the website of the Epidemiology Research Center was used to assess depression symptoms. The answers for CES-D are on a 4-scale metric, ranging from rarely to some days (1–2 days), to occasionally (3–4 days), to most of the time (5–7 days). Scores from 0 for rarely to 3 for most of the time are used for negative questions such as “Do you feel sad?” For positive questions such as “Do you feel happy?”, the scoring is reversed from 0 for most of the time to 3 for rarely. The higher the score, the more severe the depressive symptoms. Blood sample analysis was conducted in two stages. Initially, a complete blood cell count was performed at the local county health center right after sample collection. The samples were then shipped back to the research headquarters for the analysis of other biomarkers. The participants' physical examinations were conducted using professional equipment demonstrated by a measuring officer. Blood pressure was measured using the Omron HEM-7200 monitor, and the average of three systolic and diastolic pressure readings was recorded. Respiratory function was measured using a peak flow meter three times, and the average value was taken. Handgrip strength was measured twice for each hand using a dynamometer, and the average value was taken. Standing balance was determined using a stopwatch to determine whether the participant could stand with their feet together for 10 s. Walking speed was measured twice using a stopwatch, and the average value was taken. Body mass index was calculated based on height and weight. The upper arm length and knee length were measured using the MA DING ruler. Waist circumference was measured using a soft tape measure.

### 2.3. Statistical analysis

#### 2.3.1. Data pre-processing

In the dataset used in this study, some variables contain missing values, but the proportion is extremely low (<0.15%). Therefore, we used multiple imputation via regression models using the R package mice to impute the missing values. The number of disabled samples accounts for 23.22% of all participants, and as they tend to cluster into a group, it can lead to a decrease in classifier performance. To address this class imbalance issue, we utilized SMOTE (sampling technique), which oversamples the minority class by generating synthetic samples through a linear combination of existing minority class neighbors (34). We split the processed data into a 70% training set and a 30% testing set. In the dataset used in this study, count data are presented as both numerical values and proportions and analyzed using the chi-squared test. Given that the continuous data are not normally distributed, we represented it using the median and interquartile range and compared it using the Mann–Whitney U test. The training set was used for model development, while the testing set was used for adjusting model parameters and estimating the generalizability of the model.

#### 2.3.2. Model construction and evaluation

All analyses were conducted using R version 4.3.0. A *p*-value of <0.05 was considered statistically significant. Before modeling, we normalized the training and testing sets separately to prevent data leakage. The testing set was used for adjusting model parameters to avoid overfitting and for final model evaluation. The steps for model building and evaluation were as follows: (1) The least absolute shrinkage and selection operator (LASSO) regularization was employed on the training set to select significant features from the initial set of 43 variables. The algorithm underwent 10-fold cross-validation to enhance reliability. (2) Six distinct machine learning techniques, including logistic regression (LR), k-nearest neighbors (KNN), naive Bayes (NB), multilayer perceptron (MLP), random forest (RF), and extreme gradient boosting (XGBoost), were chosen to construct models. The optimization of models was performed using 10-fold cross-validation with 5 repetitions and grid searching to adjust hyperparameters, ensuring stability. (3) The performance of the models was compared using metrics such as the area under the curve, accuracy, sensitivity, specificity, and F1 score. The Youden index was used to select the threshold, and the Delong test was used to compare the ROC curves of the training and testing sets to avoid overfitting. Decision curve analysis (DCA) was performed on the testing set to evaluate the value and relative superiority of each model in the applied scenario.

#### 2.3.3. Model interpretation

**Figure 1:**
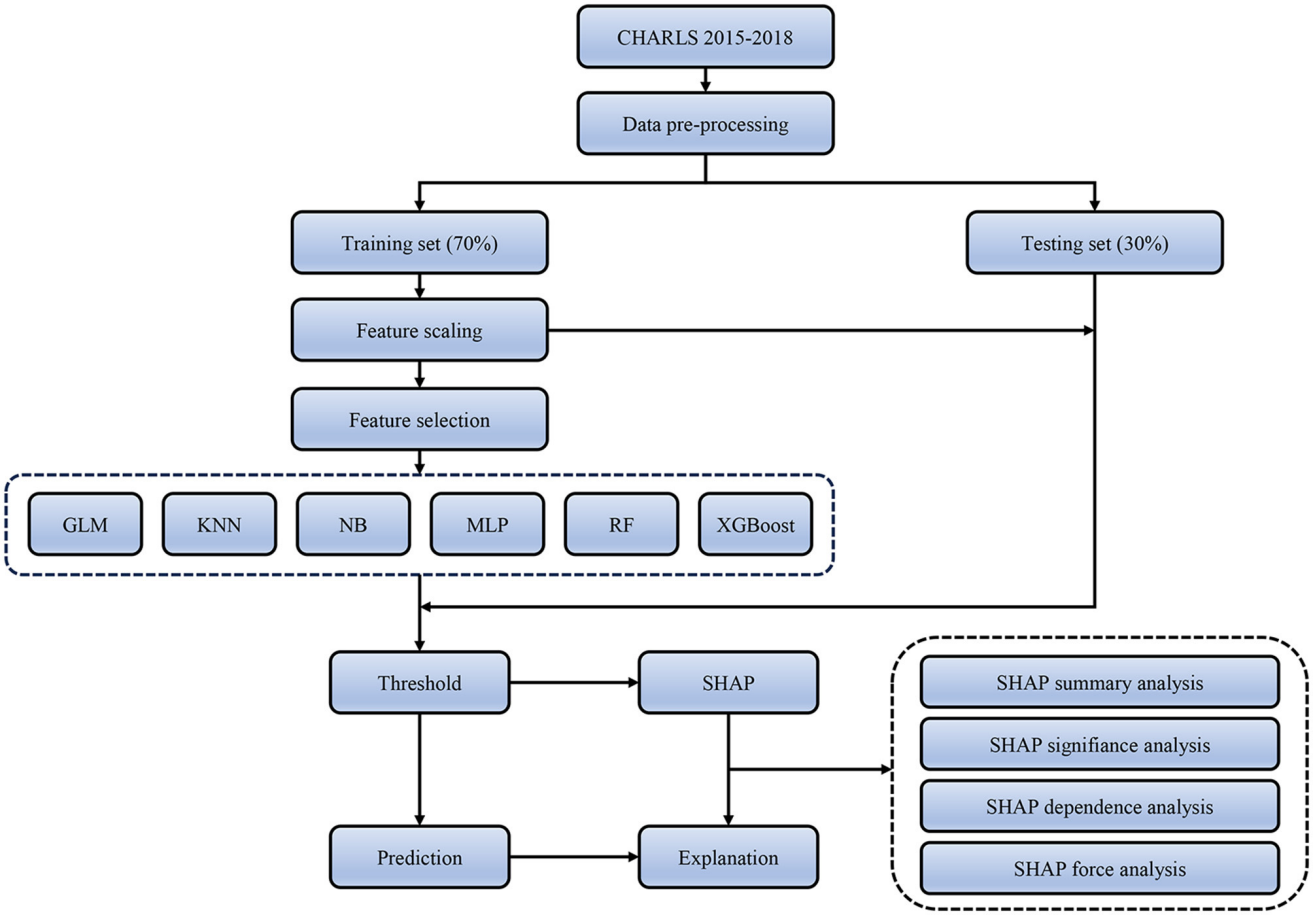
Flow chart.

The interpretability of machine learning has always been a challenging problem. To further explain how each feature variable affects and contributes to the final model, we employed the SHapley Additive eXplanation (SHAP) method to interpret the black box model with the best performance. SHAP values estimate the effect of each feature on the prediction outcome based on game theory, where each feature is considered a participant. SHAP attributes prediction performance fairly to each feature, thereby explaining each feature's contribution to a single observation. We evaluated the importance of each feature by computing the mean absolute value of its SHAP value. We also plotted the SHAP values for each feature of each sample to better understand the overall pattern and the impact range of features on the dataset. We also utilized the SHAP dependency plot to evaluate the non-linear effects of the features. Additionally, we provided two examples of SHAP predictions for the purpose of demonstration. Figure 1 displays the entire workflow of this study.

## 3. Results

### 3.1. Data processing results

This study included a population of 2,175 older adults who were in good health. Following a 3-year follow-up, we discovered that 505 of these individuals had become disabled, reflecting a disability rate of 23.22%. Owing to the imbalance of data between the disabled and non-disabled groups, we employed the oversampling process of synthetic minority classes. We were left with 3,535 samples following the application of SMOTE. The number of individuals with disabilities was found to be 1,515, accounting for 42.86%. The data were split into a ratio of 7:3, with the training set composed of 2,474 cases, of which, 1,063 were disability cases, and the testing set containing 1,061 participants, including 452 individuals with disabilities. The baseline data for both the final training and testing sets are presented in Table 1. Except for smoking, there were no significant differences in baseline characteristics between the two groups (*P* > 0.05). This suggests that the two groups were not biased due to the uneven distribution of dependent variables.

**Table 1:** Baseline characteristics in the training and testing cohorts.

**Variable**	**Training set (*n* = 2474)**	**Testing set (*n* = 1061)**	***p-*value**
Age	66.00 [63.00, 70.00]	66.00 [62.00, 70.00]	0.490
**Sex**			
Male	1, 398 (56.51)	588 (55.42)	0.575
Female	1, 076 (43.49)	473 (44.58)	
**Marital status**			
Married/cohabiting	1, 963 (79.35)	841 (79.26)	0.993
Widowed/never married/divorced	511 (20.65)	220 (20.74)	
**Education level**			
Middle school and below	2, 219 (89.69)	936 (88.22)	0.216
High school and above	255 (10.31)	125 (11.78)	
**Smoking**			
No	1, 254 (50.69)	496 (46.75)	0.035
Yes	1, 220 (49.31)	565 (53.25)	
**Drinking**			
No	1, 030 (41.63)	427 (40.25)	0.465
Yes	1, 444 (58.37)	634 (59.75)	
Sleep duration	6.70 [5.32, 8.00]	6.90 [5.40, 8.00]	0.853
**Naps**			
No	1, 040 (42.04)	438 (41.28)	0.704
Yes	1, 434 (57.96)	623 (58.72)	
Social involvement	1.00 [0.00, 1.00]	1.00 [0.00, 2.00]	0.361
WBC (1000)	5.82 [4.89, 6.85]	5.70 [4.82, 6.80]	0.137
HGB (g/dl)	13.80 [12.80, 14.80]	13.80 [12.80, 14.60]	0.318
HCT (%)	41.80 [39.00, 44.80]	41.80 [38.90, 44.60]	0.582
MCV (fl)	92.90 [89.20, 96.10]	92.80 [88.80, 96.20]	0.524
PLT (10^9^/L)	194.00 [160.00, 233.00]	193.00 [156.00, 233.00]	0.398
TG (mg/dl)	112.16 [83.19, 160.50]	113.27 [84.55, 167.26]	0.281
CREA (mg/dl)	0.79 [0.69, 0.92]	0.79 [0.69, 0.91]	0.434
BUN (mg/dl)	15.13 [12.77, 18.29]	15.41 [12.89, 18.21]	0.720
HDL (mg/dl)	50.23 [43.63, 57.14]	49.81 [43.63, 57.53]	0.658
LDL (mg/dl)	102.58 [84.31, 120.14]	101.16 [84.17, 118.92]	0.406
CHO (mg/dl)	182.63 [161.85, 203.06]	180.70 [161.78, 203.09]	0.728
GLU (mg/dl)	97.30 [90.09, 108.11]	97.31 [90.09, 111.71]	0.500
UA (mg/dl)	5.00 [4.20, 5.90]	5.00 [4.10, 5.90]	0.999
CYSC (mg/l)	0.88 [0.78, 0.97]	0.88 [0.77, 0.97]	0.777
CRP (mg/l)	1.50 [0.90, 2.70]	1.40 [0.80, 2.60]	0.189
HBALC (%)	5.90 [5.60, 6.20]	5.90 [5.60, 6.20]	0.316
Systolic blood pressure	131.00 [119.00, 144.00]	131.00 [119.00, 143.00]	0.620
Diastolic blood pressure	75.00 [69.00, 82.00]	75.00 [68.00, 82.00]	0.273
Pulse	72.00 [66.00, 79.00]	73.00 [67.00, 80.00]	0.337
Respiratory function	271.00 [203.00, 347.00]	276.00 [202.00, 350.00]	0.612
Left-hand grip strength	27.26 [22.30, 33.30]	27.00 [22.35, 33.42]	0.678
Right-hand grip strength	28.50 [23.11, 35.00]	28.60 [23.22, 35.00]	0.749
**Standing balance**			
Bad	561 (22.68)	218 (20.55)	0.175
Good	1913 (77.32)	843 (79.45)	
Walking speed	3.06 [2.60, 3.65]	3.06 [2.59, 3.63]	0.925
BMI	23.32 [21.32, 25.48]	23.40 [21.25, 25.65]	0.550
Upper arm length	33.60 [32.20, 35.00]	33.40 [32.20, 34.80]	0.356
Knee length	47.80 [46.10, 49.70]	47.60 [46.00, 49.70]	0.362
Waist circumference	86.00 [79.60, 92.77]	86.30 [79.20, 93.40]	0.655
Health satisfaction	3.00 [2.00, 3.00]	3.00 [2.00, 3.00]	0.503
Marriage satisfaction	3.00 [2.00, 3.00]	3.00 [2.00, 3.00]	0.975
Children satisfaction	2.00 [2.00, 3.00]	2.00 [2.00, 3.00]	0.352
Life satisfaction	3.00 [2.00, 3.00]	3.00 [2.00, 3.00]	0.662
Memory	4.00 [4.00, 5.00]	4.00 [4.00, 5.00]	0.249
Depression symptoms	5.00 [3.00, 9.00]	5.00 [3.00, 9.00]	0.962

WBC, white blood cell; HGB, hemoglobin; HCT, hematocrit; MCV, mean corpuscular volume; PLT, platelets; TG, triglycerides; CREA, creatinine; BUN, blood urea nitrogen; HDL, high-density lipoprotein cholesterol; LDL, low-density lipoprotein cholesterol; CHO, total cholesterol; GLU, glucose; UA, uric acid; CYSC, cystatin C; CRP, C-reactive protein; HBALC, glycated hemoglobin; BMI, body mass index.

### 3.2. Feature selection

**Figure 2:**
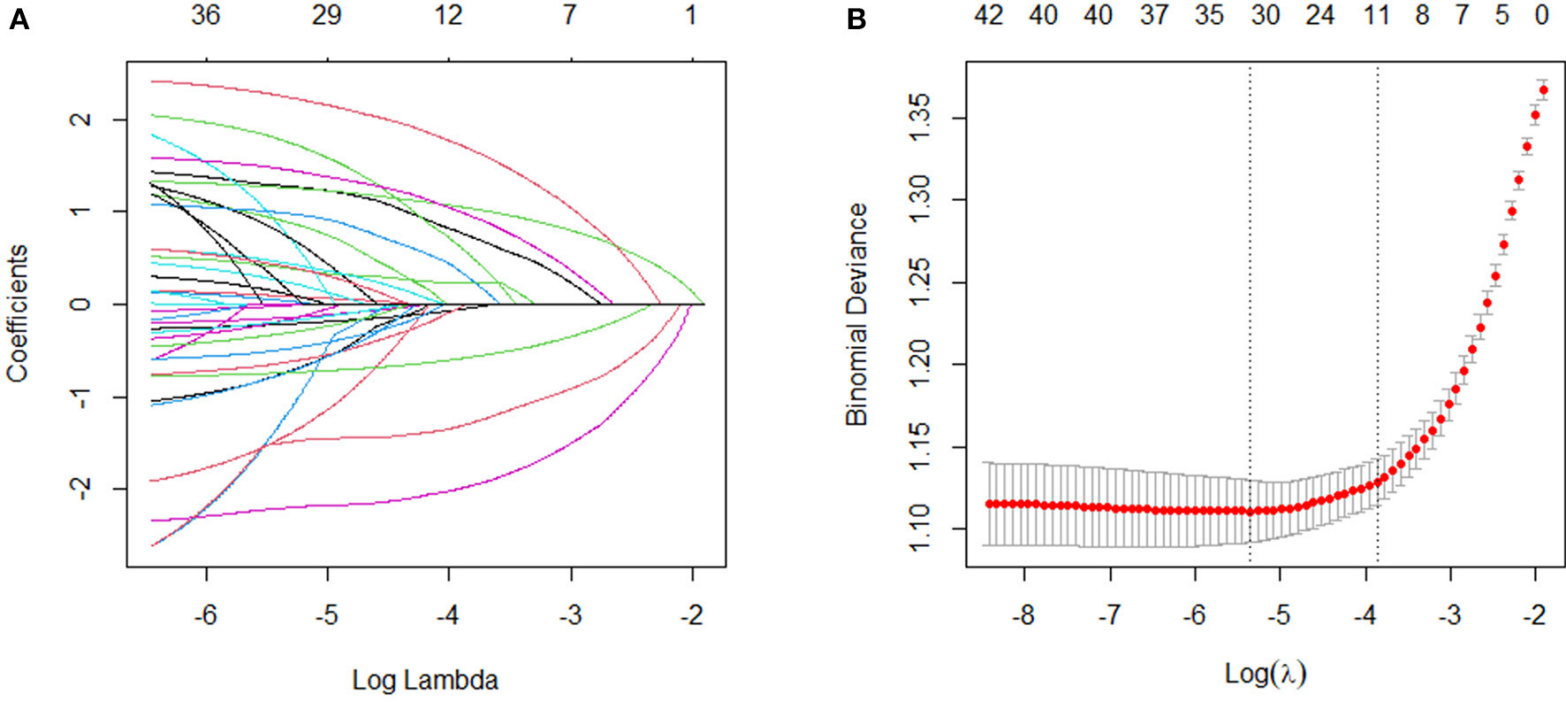
Variable selection by the LASSO regression model. **(A)** Choice of the optimal parameter (λ) in the LASSO regression model with logλ as the horizontal coordinate and regression coefficients as the vertical coordinate; **(B)** plot of λ vs. number of variables with logλ as the bottom horizontal coordinate, binomial deviance as the vertical coordinate, and number of variables as the top horizontal coordinate.

To identify the variables most strongly associated with disabilities, we normalized the training dataset to eliminate the influence of different units of measurement across the independent variables. Disability was used as the dependent variable, and the compressive variable coefficient was used to prevent overfitting and address issues of severe multicollinearity (see Figure 2A). We employed 10-fold cross-validation to determine the optimal penalty parameter, λ. We evaluated the predictive performance of the fitted models by computing the binomial deviance for the test data. The R package generates two λ values automatically—one that minimizes the binomial deviance and the other representing the largest λ within 1 standard deviation of the minimum binomial deviance. We chose the latter value of λ as it results in stricter penalties, allowing us to further reduce the number of independent variables compared with the former (see Figure 2B). Finally, we included age, marital status, naps, white blood cells, systolic blood pressure, diastolic blood pressure, respiratory function, right-hand grip strength, standing balance, memory, and depression symptoms as predictive variables to develop this machine learning model.

### 3.3. Model evaluation and comparison

According to the results of the LASSO feature selection, we attempted to use several widely used machine learning algorithms, including LR, KNN, NB, MLP, RF, and XGBoost, to construct prediction models from the training set. During the modeling process, we repeated 5 rounds of 10-fold cross-validation and grid search parameter optimization. This was performed to ensure that the models were not overfitting and had good generalization ability.

**Figure 3:**
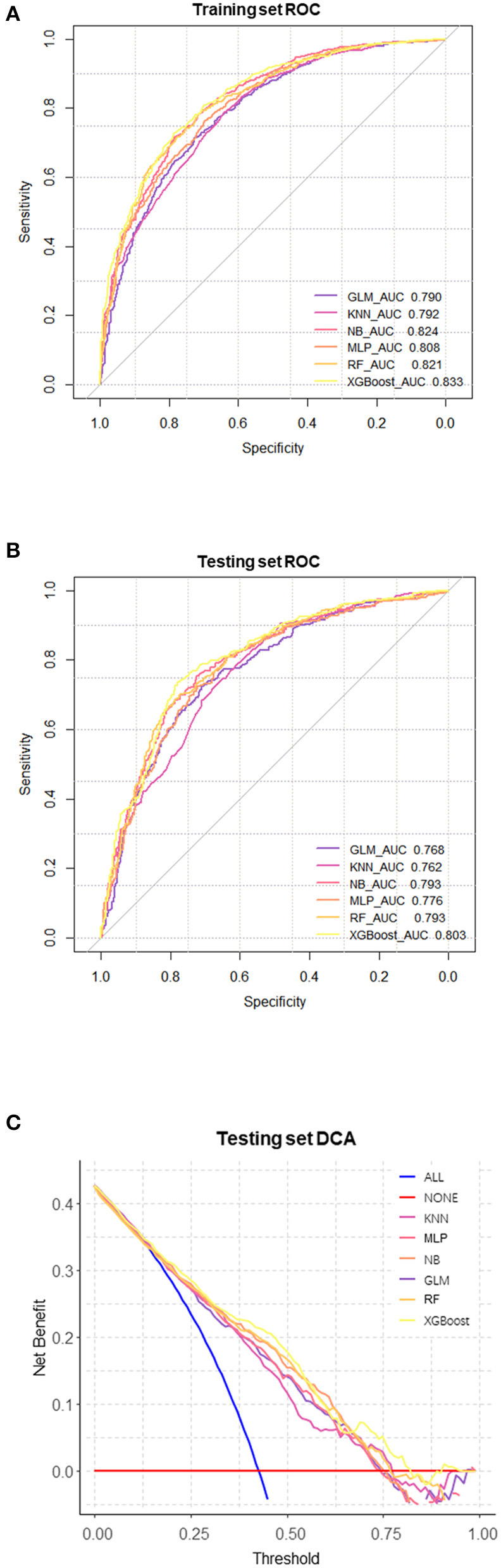
Comprehensive evaluation of machine learning models. **(A)** ROC and AUC of the training set; **(B)** ROC and AUC of the testing set; **(C)** In the testing set, the ALL curve represents the benefit rates for all cases with intervention, while the NONE curve represents the benefit rates for all cases without intervention. The remaining curves denote various models.

We evaluated the predictive models using the receiver operating characteristic (ROC) curve, accuracy, sensitivity, specificity, and F1 score. Prior to the evaluation, the optimal cutoff point was determined by maximizing the Youden index (i.e., sensitivity + specificity – 1) on the ROC curve in the training set. The results showed that the XGBoost model had the highest area under the curve (AUC) on both the training and testing sets (Figures 3A, B). In addition, except for the specificity metric, the XGBoost model outperformed other models in terms of the other performance metrics. The details of the specific model parameters developed using different algorithms are shown in Table 2. Although a high area under the curve (AUC) represents high model prediction accuracy, it is insufficient to evaluate the practical value of the model. To compare the practical utility of different models, we used decision curve analysis (DCA) to plot curves on the testing set. The DCA results (Figure 3C) showed that XGBoost had the highest net benefit in most of the threshold ranges. Taken together, these results demonstrate that XGBoost is the optimal model to use.

**Table 2:** Evaluation of the performance of the six algorithms.

**Algorithm**	**Data set**	**Threshold**	**AUC**	**Accuracy**	**Sensitivity**	**Specificity**	**F1**
LR	Train	0.423	0.790	0.721	0.727	0.713	0.748
LR	Test	0.423	0.768	0.714	0.718	0.708	0.742
NB	Train	0.560	0.824	0.756	0.786	0.716	0.786
NB	Test	0.560	0.793	0.739	0.778	0.686	0.774
KNN	Train	0.432	0.792	0.705	0.644	0.787	0.713
KNN	Test	0.432	0.762	0.689	0.629	0.770	0.699
MLP	Train	0.371	0.808	0.726	0.698	0.763	0.744
MLP	Test	0.371	0.776	0.716	0.704	0.732	0.740
RF	Train	0.339	0.821	0.744	0.714	0.785	0.761
RF	Test	0.339	0.793	0.715	0.683	0.759	0.73
XGBoost	Train	0.478	0.833	0.761	0.787	0.726	0.790
XGBoost	Test	0.478	0.803	0.757	0.790	0,712	0.789

### 3.4. Model interpretation

**Figure 4:**
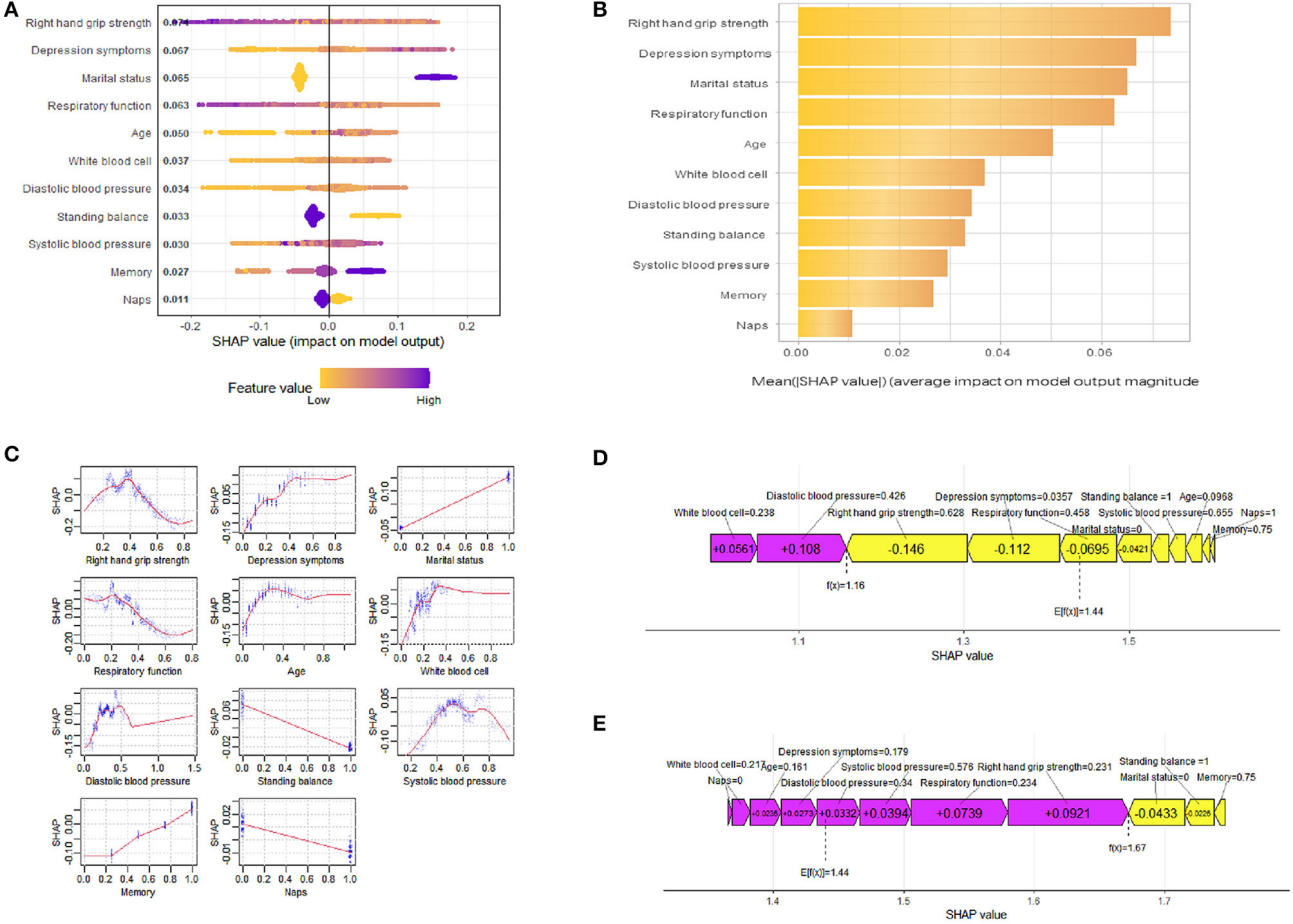
SHAP interprets the model. **(A)** All samples and features are illustrated, with each row representing a feature and x-axis representing the SHAP value. The yellow dots represent higher feature values, while the purple dots represent lower feature values. **(B)** Ranking of variable importance based on the average value. **(C)** The SHAP dependence plot of the XGBoost model. **(D)** SHAP predictions for no disability samples. **(E)** SHAP predictions for disability samples. Purple arrows indicate a higher risk of disability, while yellow arrows indicate a lower risk of disability. The length of the arrows helps visualize the degree of impact of the prediction, whereby the longer the arrow, the more significant the effect.

To better understand the relationship between the model and the data, we provide a more intuitive interpretation of the best-performing XGBoost model using SHAP to illustrate how these variables affect the 3-year disability rate in the model. Figure 4A illustrates the 11 evaluated risk factors by their SHAP values. The SHAP value, located on the x-axis, is a unified index that determines how a certain feature affects the model's outcome. In each feature important row, participants' attributions to the outcome were drawn with colored dots of high- and low-risk values represented by purple and yellow dots, respectively. Figure 4B displays the important features in this model where the feature ranking, located on the y-axis, indicates the predictive model's significance. The findings show that there is a high correlation between right-hand grip strength, depression symptoms, marital status, respiratory function, age, and the 3-year disability prediction probability in healthy older adults. The SHAP dependence plot (Figure 4C) can also be used to understand how a single feature affects the output of the XGBoost prediction model. Additionally, we provide two typical examples, one predicting no disability (Figure 4D) and the other predicting disability (Figure 4E), to demonstrate the model's interpretability.

## 4. Discussion

In this retrospective cohort study, we established a predictive model to estimate the 3-year risk of disability in older adults aged 60 and above in China who had good health status. We employed machine learning algorithms for the prediction task and utilized the LASSO method for feature selection; six machine learning algorithms were deployed for the prediction task and ultimately developed and validated the model using 11 important features. Among the tested models, the XGBoost model performed the best in terms of predictive performance. By analyzing the best-performing model using SHAP, we identified influential features such as right-hand grip strength, depressive symptoms, marital status, respiratory function, and age. Additionally, we demonstrated how every feature affected the model's disability predictions.

From the point of view of influence factors, the selection of features or variables is crucial in developing prediction models (35). Among the initial 43 variables, the LASSO algorithm assisted in identifying 11 significant variables. These variables support previous literature regarding demographic characteristics (36), depressive symptoms (37), and physical examinations (38, 39) in older adults, highlighting the reliability and relevance of the chosen predictors. In this study, grip strength emerged as a predictor of disability in older adults. Grip strength provides a convenient assessment of overall strength capacity, which is associated with various health conditions. Research indicates that individuals with lower grip strength have a 1.42 times higher risk of disability compared to those with symmetrical and strong grip strength. Moreover, individuals with asymmetric and weak grip strength face an even higher risk of up to 1.86 times (40). Researchers have found that lower grip strength could be a significant risk factor for ADL disability in older adults (41). Compared with other common factors associated with physical frailty, such as comorbidities, weight loss, or fatigue, grip strength is highly predictive of ADL disability (42). The strength of grip is directly related to muscle strength, which plays a key role in executing various actions and daily activities. The assessment of grip strength is closely associated with changes in skeletal muscle strength and alterations in physical functioning (43). A decline in grip strength may indicate a decrease in the functional capacity of relevant muscle groups, affecting an individual's physical function and activity ability, thereby increasing the risk of disability. We found that depressive symptoms are a key predictor of disability in older adults. This finding is consistent with previous research (44–46), which demonstrates a longitudinal association between depressive symptoms and disability in older adults. Over time, individuals with higher baseline depression levels are more likely to report difficulties in ADL abilities. Physiologically, depressive symptoms can act as a stressor that triggers and exacerbates inflammatory processes, thereby increasing the subsequent risk of disability. Moreover, physical symptoms associated with depression, such as fatigue and pain, may contribute to a decline in physical functioning among older adults, impairing their ADL abilities (47). From a psychological perspective, older adults experiencing depression are more likely to lose hope in life, resulting in a reduced likelihood of adhering to long-term medical advice (48). Additionally, they may lack motivation for social and outdoor activities, losing the protective effects of social engagement on health and further weakening ADL abilities (49). Marital status is an important predictor of disability in older adults. Among all marital statuses, never-married and widowed older adults report the highest incidence of disability compared with other groups (50). Spousal support plays a unique role in the health of the Chinese population, indicating a significantly increased disability risk in older adults who are lifelong single or widowed (51). This can be attributed to the positive influence of spousal companionship on health behavior management and the healthcare environment for older adults. Married individuals are more likely to adopt healthy lifestyles, seek higher quality healthcare, and invest more in healthcare expenditure (52). While the majority of older adults in the cohort were married, the increasing rates of staying unmarried and divorced in the current society necessitate further research on marital status and its impact on disability. Respiratory function is a strong predictor of disability in older adults. Previous research suggests that, for every 1-unit decrease in respiratory function, the risk of developing mobility limitations increases by 1.6 times (53). Respiratory function is controlled by different distributed neural networks, starting from the brain and extending to peripheral muscles. Pathological features at multiple levels throughout the neural axis may result in weakened respiratory function, causing mobility limitations and increasing the risks of ADL and IADL disabilities (54, 55). Although previous literature has reported a relationship between respiratory function and disability, it has not been included as a predictor in existing disability prediction models. The study results indicate that older adults with stronger respiratory function have a lower risk of disability, while those with weaker respiratory function have a higher risk. This highlights the potential importance of respiratory function as a novel predictor. Age is also associated with the risk of disability in older adults. A study by Chinese scholars on the trends of disability in Chinese older adults over a 10-year period found that ADL disability increases with age, and compared to period effects and cohort effects, the age effect is the strongest (56). Another survey of older adults aged 65 years and above revealed that bathing and dressing/undressing were the most prevalent ADL disabilities, with a higher proportion of participants experiencing IADL disabilities (31.9%). The ≥85 age group exhibited the highest prevalence of both ADL and IADL disabilities compared with other age groups (32). While the likelihood of disability may vary due to social and population differences among different countries, the increase in disability risk with age in older adults seems undeniable. Although the influence of the following factors on disability is not as significant as the aforementioned predictors, we also found higher WBC levels, an inability to maintain static balance, poorer memory, and a lack of siesta to be risk factors for disability in older adults. Blood pressure is also one of the influencing factors, but according to the results, it does not exhibit a significant linear trend in its effect on disability. Furthermore, we emphasize that all the predictors included in this study can be measured in the real world. In particular, physical examinations, as an integral part of the final model, provide more objective and stable information compared with self-reported measures.

From a model construction perspective, previous studies have reported several risk prediction models for disability, with most of them using the COX proportional hazards model and multivariable logistic regression analysis (57–60). Although only a few studies have used machine learning algorithms for predicting disability risk in older adults, machine learning models have been shown to outperform traditional models in disability prediction (17, 61, 62). During the process of constructing disability risk prediction models, we encountered several important issues. First, imbalanced data can significantly affect model performance in the field of biomedicine (63, 64). In the older population, the relatively low disability rates lead to an imbalance in the proportion of normal and disabled samples. For instance, if the disability rate among older adults is 20%, even if the model predicts all results as normal, there would still be an accuracy of 0.8, which is clearly incorrect. This imbalance also leads to a tendency of the model to predict normal samples more frequently and subsequently shows less accuracy in predicting disabled samples. However, the aforementioned studies did not adequately consider this point during model construction. In machine learning, methods such as oversampling or under sampling are recommended for addressing the issue of imbalanced data (65). Therefore, we adopted the SMOTE sampling technique to balance the normal and disabled samples, thereby improving the prediction accuracy and stability of the model by balancing the number of older adults in different categories in the dataset. Second, regarding variable selection, a 5-year follow-up study on disability prediction in older adults from Japan suggested that a prediction model constructed solely using self-reported variables could predict functional impairments with good performance (61). However, age explained 50–70% of the predictive performance in their best model, indicating the limited predictive value of other features. As older adults are a group with rapidly changing health conditions, relying heavily on age for prediction would be unreasonable, considering the high incidence of disability within 5 years. Some studies have also used ADL as one of the predictive factors (66). The functional impairments in older adults are difficult to recover, and the likelihood of returning to normalcy toward the end of follow-up is extremely low for individuals who already have baseline impairments. Whether daily activity ability can serve as a predictive factor for disability remains to be further studied. Given the complex factors influencing disability, it is worth considering more variables and using techniques such as regularization for selection. Finally, model performance evaluation is also a challenge. The AUC is the most widely used metric. In a study on daily living disability prediction in a Chinese older community population from the same cohort similar to our study cohort, researchers constructed six models based on different physical performances, with AUCs ranging from 0.693 to 0.718 (67). In this study, the AUCs of the six machine learning algorithms ranged from 0.790 to 0.833, indicating that our research could provide a reference for improving disability prediction models for Chinese older adults. Overfitting is another issue to be considered in model evaluation (68). In the modeling process, even with methods such as cross-validation, some models still exhibited very high AUC values and achieved high accuracy even on test data. However, statistical tests revealed differences in AUC between the two datasets, suggesting that the generalization ability of models trained only on internal validation is questionable. Therefore, without completely independent external validation data, we recommend splitting a portion of the data before normalization and variable selection and treating it separately for mimicking external validation and parameter adjustment to control model overfitting. For the construction and validation of more standardized models in medicine, relevant guidelines and studies can be consulted (69–71). In summary, although we faced challenges in constructing disability risk prediction models, machine learning algorithms have the potential to address these issues. By addressing data imbalance, selecting relevant variables effectively, improving model accuracy, and controlling overfitting, we established a Chinese disability prediction model for healthy older adults with high predictive and generalization ability, providing more accurate and reliable guidance for health management and disability risk interventions for Chinese older adults.

From a model interpretation perspective, traditional machine learning algorithms are often criticized for lacking transparency and interpretability (72, 73). In order to better understand the inherent logic and decision rules behind model predictions, another advantage of this study was the use of SHAP values to interpret these machine learning models and reveal their black box issues. XGBoost, the best-performing model, was the one we focused on for interpretation. In these test data, we calculated the SHAP values for each feature variable to assess their contribution to the prediction results. The overall SHAP summary plot helps us understand which features positively and negatively affect the prediction results, while the importance feature plot provides an average assessment of the feature importance for the entire dataset. This graph helps us understand the contribution of each feature to the prediction results for this population and provides useful information for further analysis and interpretation of the model. Additionally, the SHAP dependency plot helps to observe how these features affect the output of the prediction model at different levels. In this study, the influence of marital status, balance ability, memory, and napping on results can be clearly observed. Grip strength, depressive symptoms, respiratory function, age, and WBC count not only show a certain linear relationship with the prediction results but also exhibit some turning points that warrant further investigation. For example, age initially increases the risk prediction of disability, followed by a slight decrease. This may be because the mortality rate among older adults is higher in a certain age group, and the surviving older adults tend to exhibit relatively better health conditions, which cannot be observed in linear models. Systolic and diastolic blood pressure also plays important roles in predicting disability risk in older adults but do not show obvious linear relationships. In other words, the prediction results are not only influenced by the levels of each factor but also by the individual differences among them. Therefore, personalized risk prediction is also needed. The SHAP force plot provides examples of how different features contribute to individual risk prediction. In positively predicted samples, grip strength, respiratory function, and marital status are the most influential factors in the results. In negatively predicted samples, grip strength, depressive symptoms, and diastolic blood pressure are the most influential factors. This is similar to the overall feature importance and also reflects the heterogeneity of older adults. Previous one-size-fits-all intervention policies for disability prevention have had limited effectiveness (74). Personalized risk prediction provides guidance for relevant decision-makers, such as offering more targeted health and community services for specific age groups. Overall, the SHAP values used in this study provide a method to unravel the black box of machine learning models, enhancing their interpretability and transparency. This allows us to better understand the predictive results of the XGBoost model for disability risk prediction in Chinese healthy older adults. By analyzing SHAP values, we can quantitatively evaluate the extent to which these factors influence the prediction results, identify potential risk groups among healthy older adults, and provide a basis for intervention measures and personalized prevention. This helps improve the quality of life for older adults, reduce the burden on healthcare systems, and promote healthy aging.

## 5. Limitations

There are several limitations to this study. First, there are no universally accepted inclusion and exclusion criteria for defining health. Although we established some selection conditions, this may still result in the inability to identify hidden relationships between certain individuals and outcomes. Second, the data were obtained from a nationally representative survey, which may limit its applicability and benefit thresholds in specific regions. Third, the selected variables are limited by the structure of the survey questionnaire, and thus, we cannot guarantee the inclusion of all potential factors in this study. Whether to include the selected variables as categorical or continuous, as well as the choice of the division criteria, can also have an impact on the results. Finally, despite employing certain methods to ensure the reliability and generalizability of the model, the results still need to be validated in an externally independent cohort. Despite these limitations, we believe that these findings are applicable in the prevention and further intervention of disability occurrence among healthy older adults in China.

## 6. Future routes of research

While the research results have significant implications, future ideas and avenues for exploration remain to be investigated and developed. Given the limitations of machine learning, we present potential directions for further research that may aid in advancing the field. First, we highlight the importance of diversity in both variables and models. Currently, many factors have been identified as predictive of disability occurrence, but the importance of each factor may differ among different modeling populations. To more accurately predict the risk of disability occurrence, we need to collect more comprehensive and diverse data, including information on different demographics, regions, and environmental factors. With these data, we can tailor models to specific populations, such as constructing multiple disability risk prediction models based on the age range of older adults. Multiple models can not only remove the influence of disability occurrence rates in different age groups but may also reveal unique predictive variables for each age group. Second, the selection of predictive variables is crucial in building machine learning models. At present, there are mainly three methods for variable selection, including filter methods, wrapper methods, and embedded methods. Although they have the same purpose, the evaluation criteria for selecting predictive variables and the variables selected by each method may differ. Future research can consider using all three methods to establish predictive models for comparison. Alternatively, one can take the intersection of the predictive variables selected by the three methods and construct the disability risk prediction model. Finally, there is a need to focus not just on model development but also on model interpretability. While building accurate and effective disability risk prediction models is not difficult with the continuous development of machine learning models, the focus should shift toward model interpretability. Although SHAP, as a model interpretability method, has some value, further research is needed to develop machine learning models with stronger interpretability. This would enable doctors and researchers to understand the decision-making process of the model and would ultimately benefit the population at risk of disability. In addition, machine learning predictive models may also be used for long-term monitoring of an individual's health, which is uncommon in medical research. Future research can explore ways to integrate predictive models with real-time monitoring technologies, identify potential disability risks in a timely manner, and provide personalized health intervention measures to help people improve their lifestyles and prevent disability. These are some ideas and routes for future research on disability risk prediction. With further research and innovation, we can expect more accurate and effective development of disability risk prediction models, making greater contributions to public health.

## 7. Conclusion

In conclusion, we successfully utilized machine learning methods to predict the 3-year disability risk among older adults in China. The XGBoost model demonstrated superior performance in this study. Additionally, we addressed the “black box” issue associated with machine learning by employing SHAP for explanation. SHAP not only helped determine the importance of each feature in the model but also demonstrated how each feature influenced the model. This is highly valuable for the early identification and intervention of healthy older adults with potential disability risks.

## Data availability statement

Publicly available datasets were analyzed in this study. This data can be found here: http://charls.pku.edu.cn/index.htm.

## Ethics statement

The studies involving humans were approved by Institutional Review Board at Peking University. The studies were conducted in accordance with the local legislation and institutional requirements. The participants provided their written informed consent to participate in this study.

## Author contributions

YH: Data curation, Formal analysis, Writing—original draft. SW: Supervision, Writing—review & editing.
